# Enhancing clinical reasoning for management of non-communicable diseases: virtual patient cases as a learning strategy for nurses in primary healthcare centers: a pre-post study design

**DOI:** 10.1186/s12909-024-05440-z

**Published:** 2024-04-23

**Authors:** Gerard Nyiringango, Uno Fors, Elenita Forsberg, David K. Tumusiime

**Affiliations:** 1 Department of Computer and Systems Sciences (DSV), Stockholm University, Borgarfjordsgatan 12, PO Box 7003, 164 07 Kista, Sweden; 2 Department of Nursing, School of Nursing and Midwifery, College of Medicine and Health Sciences, University of Rwanda, P.O.Box 3286, Kigali, Rwanda; 3 School of Health and Welfare, Halmstad University, Halmstad, Sweden; 4 School of Health Sciences, College of Medicine and Health Sciences, University of Rwanda, Kigali, Rwanda

**Keywords:** Virtual patient cases, Virtual case system, Continuous professional development, Nurses, Health care providers, Primary health care, Assessment, Clinical reasoning, Pre-posttest

## Abstract

**Background:**

In Rwanda, nurses manage all primary care at health centres, and therefore are their clinical reasoning skills important. In this study, a web-based software that allows the creation of virtual patient cases (VP cases) has been used for studying the possibility of using VP cases for the continuous professional development of nurses in primary health care in Rwanda. Previous studies in pre-service education have linked VP cases with the enhancement of clinical reasoning, a critical competence for nurses. This study investigated the feasibility of continuous professional development through VP cases to further train in-service nurses in clinical reasoning.

**Method:**

The study used a pre-post test design. Initially, seventy-six participants completed a questionnaire as part of the pre-test phase, subsequently invited to engage with all four VP cases, and finally responded to the post-test questionnaire evaluating clinical reasoning skills. Fifty-six participants successfully completed the entire study process and were considered in the analysis. The primary outcomes of this study were evaluated using a paired t-test for the statistical analysis.

**Results:**

The results show that the mean score of clinical reasoning increased significantly from the pre-test to the post-test for all four illness areas (*p* < 0.001). The study findings showed no statistically significant difference in participants’ scores based on demographic factors, including whether they worked in urban or rural areas.

**Conclusion and recommendation:**

Utilizing VP cases appears to significantly enhance the continuous professional development of nurses, fostering a deliberate learning process that enables them to reflect on how they manage cases and, in turn, refine their clinical reasoning skills. This study strongly recommends incorporating VP cases in the continuous professional development of nurses at the primary health level (health centers). This is especially pertinent in a context where nurses are required to perform diagnostic processes similar to those employed by physicians.

## Background

Clinical practitioners, including nurses, require continuous professional development (CPD) that helps them improve their practicing skills over time [1]. This might be even more important for less experienced clinicians. The occurrence of new diseases or new methods of managing existing diseases also emphasizes the importance of CPD for nurses. Many countries, including Rwanda, have made CPD mandatory to renew professional licenses so that clinicians can improve practice and minimize errors in providing health care services [1].

In Rwanda, nurses are responsible for making the first patient consultation at the health center level (a primary health care level) and deciding to manage or transfer patients to another level (district hospital) where a patient can meet a physician (physicians are usually not available at health centers). According to the Ministry of Health in Rwanda, 85% of the burden of disease is addressed at the primary health care level, including community, health posts, and health centers [2]. At this healthcare system level, nurses use diagnosis procedures traditionally applied by physicians to assess and manage diseases, exposing them to risks for diagnostic errors. Nurses are responsible and accountable for patient care and work to their full potential without supervision at the health center. While physicians may occasionally make diagnostic errors, "diagnostic errors persist throughout all settings of care, involve common and rare diseases, and continue to harm an unacceptable number of patients” [3], nurses at primary health care centers might be more prone to committing errors due to limited access to advanced clinical tools and limited training. Thus, it is imperative for nurses to continuously update and enhance their practical skills, including clinical reasoning.

The Ministry of Health in Rwanda employs both face-to-face and, more recently, online approaches for delivering CPD to nurses. This traditional approach to CPD primarily relies on Multiple Choice Questions (MCQs) to evaluate the learning outcomes after CPD training. This study used a different CPD approach (VP cases) to train and evaluate the outcomes. The current study focused especially on skills for early disease detection, consequently promoting appropriate management among in-service nurses working at health centers.

### Virtual patient cases

“Virtual patients are interactive computer simulations of real-life clinical scenarios for the purpose of healthcare and medical training, education, or assessment ([4] p.170). VP uses a computer or tablet screen that allows the users to conduct a complete patient history, make a physical examination, request laboratory or imaging examination, make a diagnosis, and thereafter make a management plan [5, 6]. The literature indicates that VP cases can help learners acquire communication and clinical reasoning skills, but many authors attribute the use of VP cases to especially training clinical reasoning skills [7–9]. A VP case reflects real patient case scenarios where software responds to the asked question as a patient could do and this feature provides a realistic and engaging learning environment in health education [10]. These interactive features, enable the learners to acquire clinical reasoning competencies [11]. VP cases take the learners into a similar reasoning and decision-making process they would encounter if they met actual patients [12]. These features make the VP cases the teaching tool and various health institutions have started using similar systems for pre-service training for many years [8, 13, 14].

While VPs have traditionally been used in pre-service education, this study utilizes VPs as a teaching tool for exposing nurses at health centers to a diverse range of diseases. The intention is to provide a platform for gaining valuable experience that could contribute to improved disease management skills. The use of VP in this study was inspired by Kolb’s four stages of learning: concrete experience, reflective observation, abstract conceptualization, and active experimentation [15]. In this study, using VP cases for CPD of nurses was a concrete experience; nurses observe and connect with a VP case. In reflective observation, nurses look for the meaning of the information presented in VP cases and reflect on it. In abstract conceptualization, they think and conceptualize the information, and during active experimentation, they manage the case by applying what they know and searching from different resources. The virtual patient system used in this study is a web-based software called Virtual Case System (VCS) that allows creating, editing, and running virtual patient cases (VP cases) and assessing learning outcomes through both open and closed questions.

### Clinical reasoning

Clinical reasoning is a crucial competence for healthcare providers. Clinical reasoning is "a complex cognitive process that uses formal and informal thinking strategies to gather and analyze patient information, evaluate the significance of this information, and weigh alternative actions”  ([16] p.1155). It is an important skill that enables a healthcare provider to make appropriate decisions and minimize diagnostic errors [17]. Studies indicate that VP cases can enhance clinical reasoning skills among nurses with practicing experience as well as senior students [12]. Health teaching institutions often use VP cases to teach healthcare students. However, many studies recruited those who have been practicing or students who are in the final years of their studies. For example, Forsberg et al. [11] used VP cases to assess the progression of clinical reasoning of postgraduate nurses. The findings of that study indicated a progression of clinical reasoning skills. The nurses in that study had working experience as a pre-requisite criterion for postgraduate registration. In another study, Friedman and Goldschmidt (2014) recruited students who were also practicing registered nurses (RN) and concluded that VP cases "are well suited for practicing RNs, as they require the application of knowledge, while at the same time building upon previously learned knowledge and experience" ([12] p. 282). While the literature supports the use of VP cases for clinical reasoning training among nurses with clinical experience, to our knowledge, there are limited studies on the use of VP cases for CPD of in-service nurses.

VP cases can be used for learning about almost any type of clinical case. However, this study chose non-communicable diseases (NCDs) due to their status as an emerging health challenge in the Rwandan healthcare system. NCDs are the leading cause of morbidity and mortality worldwide, and there is a growing and pressing burden of NCDs in developing countries [18]. As a developing country, Rwanda still has a large burden of infectious diseases, but NCDs are also an increasing burden to the Rwandan health system. The Ministry of Health report indicates that in Rwanda, there is a shift in disease burden where NCDs are becoming more prevalent and requests the adaptation of early screening [2]. Thus, the study randomly focused on managing NCDs of hypertension, depression, prostate cancer, and gastric cancer, specifically at the level of primary health centers. This study aimed to investigate the feasibility of continuous professional development through VP cases to further train in-service nurses in clinical reasoning.

## Methodology

### Study design

This study used a pre-post test design to assess the outcomes of the intervention [19]. The study assessed participants’ clinical reasoning before and after their engagement in an intervention involving VP cases as a CPD learning approach.

### Study intervention

Web-based VP systems for learning may have different structures according to the learning goals in focus. Before starting this study, the researchers adopted an existing web-based VP system developed at Stockholm University, called Virtual Case System (VCS), into the local context of health centers in Rwanda on how they collect patient information and make decisions. The VCS system, as well as the VP cases we used in this study, were therefore adopted to mirror the Rwandian healthcare situation, with available resources at local health centers and nurses as the primary contact in terms of illness scope, management procedures, patient information, history taking, status, lab/imaging, assessment, and feedback. In addition, researchers chose the NCDs of hypertension, depression, prostate cancer, and gastric cancer to be included in this study as VP cases. These four NCDs are common in Rwanda.

### The development of VP cases and assessment tools

The development of the VP cases for training progressed alongside the creation of the pre/post-test paper cases (assessment tools) and their corresponding responses. This process proceeded through the following steps:

Step one: the first author extracted relevant cases from real patient files and removed all identifier information. Step two: the research team reviewed and agreed on the first draft of VP cases and assessment tools that should be used in the study. Step three: the reviewed VP cases designed by the research team were sent to local nursing experts for validity review. The potential questions (assessment tools) and their corresponding responses, utilized in the learning assessment, were also sent alongside the VP cases to the expert team. The expert team consisted of experienced nurses who supervise the practice of nurses at health centers and teach nurses at the University level in Rwanda. The expert in prostate cancer/benign prostatic hyperplasia was a nephrology nurse practitioner and educator. The expert on gastric cancer was an oncologist, nurse practitioner, and educator. For experts in hypertension, we consulted two medical-surgical nurse practitioners and educators, and as experts on depression, two mental health nurse practitioners and educators were engaged. In step four of developing VP cases and assessment tools, the research team addressed comments and suggestions from the experts. Step five: the adapted cases and assessment tools were sent back to the experts to request if they were satisfied with the way their inputs were included in the VP cases and assessment tools. Step six: the VP cases and assessment tools were piloted on three nurses who work at health centers to verify that the cases, and assessment tools, were as good as possible. Finally, the VP cases and assessment tools were approved by the research team and a team of experts in a final workshop after adopting the VP cases, and assessment tools to pilot findings. At this point, all cases were entered into the VCS system and were decided to be ready for the CPD test. The assessment tool was also ready to be used for assessment (refer to the explanation of assessment tools on page 11).

**Fig. 1:**
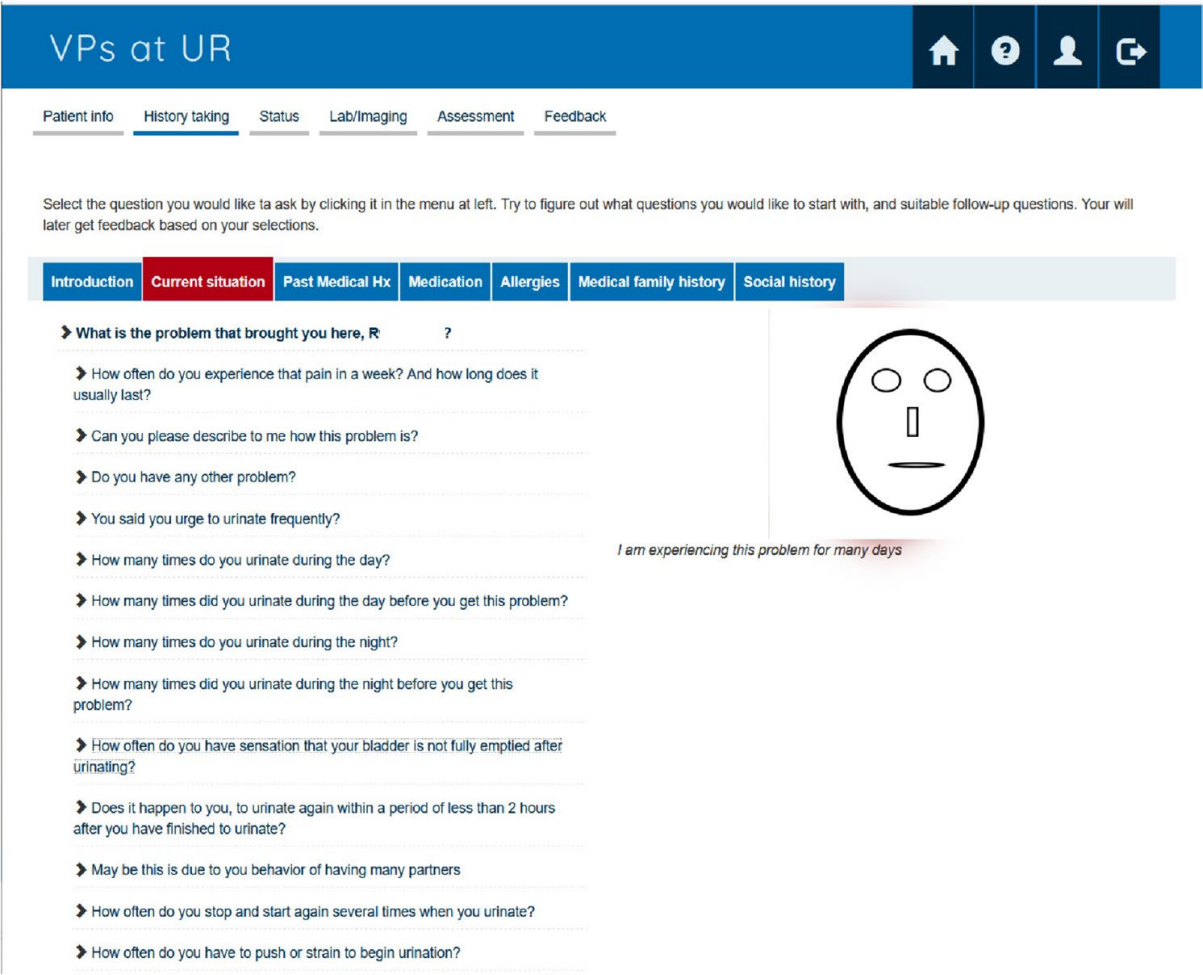
Screenshot of the illness history section of VCS (the schematic virtual patient drawing only for illustration purpose in this paper; in the running system it shows a photo of a human being)

**Fig. 2:**
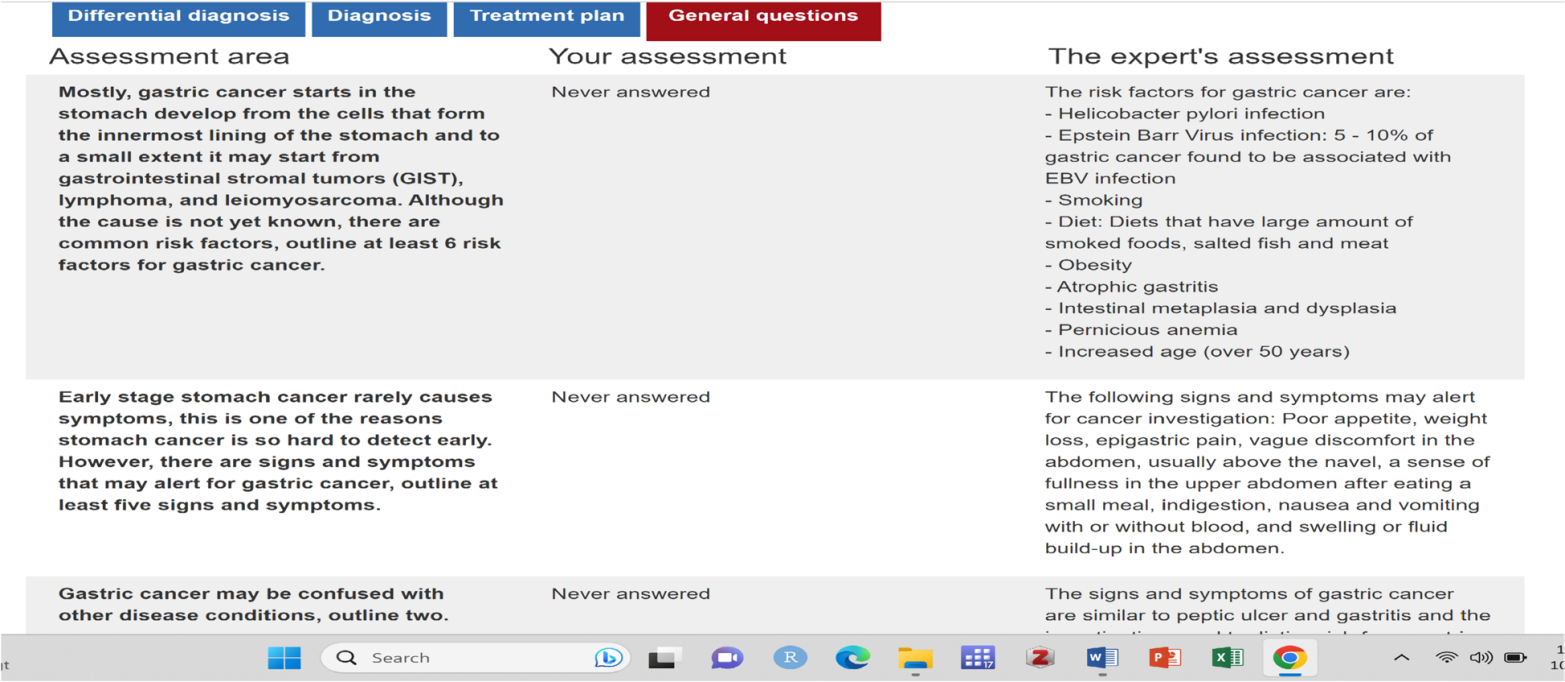
Screenshot of one of the feedback pages from VCS

The cases in VCS allowed the users to ask questions to the virtual patient, order a limited number of lab tests, and answer questions on managing the actual case, see Fig. 1. After answering the questions, the feedback section is opened; see Fig. 2.

### Explanation of assessment tools

To assess skill levels of the participating nurses, paper based cases were applied as pre-and post-tests. The study used the experience of traditional case study development in nursing schools in Rwanda (which often are based on paper-based case descriptions). The questions and answers of the paper cases were also validated through the research team discussions, input from experts, and adapting to pilot study findings. Each paper case starts with a patient narrating how she/he feels and what problems she/he has, and then the participant (nurse) is asked what other illness history question(s) should be of value to manage the case. Next, the paper case revealed more information that allows the nurse to request/suggest/think about physical and/or laboratory investigations. The nurse was then requested to suggest a necessary physical and/or laboratory exam according to the patient's condition. The narrative then proceeds to reveal more information in the next part of the paper case, including the physical and laboratory exam results. The nurse was finally requested to propose proper management of each paper case.

Each question was on a separate page. The participant started with the first page and was given the next page after he/she had submitted the previous page. The participants were not allowed to go back and change answers on the previous page(s). The research team and experts also developed a list of possible answers to each question asked, used for the subsequent correction of the paper cases.

For example, the paper case questions for gastric cancer were similar to the following (each question was written on a separate page):

1. Mr. R (this is not the real name of a patient), aged 67, has come to the health centre complaining of abdominal pain that has lasted for a long time. What are the five most important questions you can ask Mr R during history taking about his chief complaints?
2. The sickness history of Mr. R revealed that he was taking antacids for heartburn medication for abdominal pain, which is no longer responding. He also reported having signs of indigestion, vomiting, and passing of blood stool. Vital signs of Mr. R are as follows: pulse: 86 beats/minute, BP: 107/91 mmHg, oxygen saturation: 95%, temperature: 36.4 °C. Are there any laboratory examinations (available at the health centre level) that you should request for Mr. R? If yes, what are those laboratory examinations?
3. All laboratory examinations requested at the level of a health centre for Mr. R were negative, or you did not request any laboratory examination. What four most likely differential diagnoses (all possible diagnoses) can you consider for Mr. R?
4. After you have made a differential diagnosis, give one diagnosis you think is the most probable for Mr. R.
5. According to your most likely diagnosis, which two most appropriate management/procedures (treatment, medication, referral, or similar) can you give or advise Mr. R?

All four illness areas were assessed in this format (before and after the intervention).

Each case was assessed depending on the answers to the paper case, where 0 was the minimum score, and 15 was the maximum score per paper case. After data collection, all answer sheets from the participants were made into an extra copy to allow the awarding of scores by two different nurses per paper case. Since all questions were open-ended, interpreting the answers differently was possible. Thus, two nurses marked the same answer sheet separately and awarded scores separately. A third nurse compiled the marks and registered the average from the scores awarded by the two nurses. The sheets were marked based on pre-determined answers. Any answer not on a pre-determined list of answers was not considered in grading. However, a participant could have written an idea using his/her own words. In that case, the answer was graded as the usual practice of grading case studies of nurses in Rwanda.

### Sample size calculation

To determine the appropriate sample size, G*power 3.1 was used [20]. The sample size calculation considered the paired t-test (difference between two dependent means) used to test participants' clinical reasoning before and after participating in CPD using VP cases. Thus, an alpha of 0.05, a power level of 0.95, and an effect size of 0.5 were used to determine the sample size. Based on the G*Power calculation, the sample size required for this study was 45 participants. Given the prospective nature of the study, we anticipated a significant attrition rate and therefore aimed to recruit twice the number of participants in the pre-test phase.

### Study setting and sampling strategies

This study was carried out in Rwanda. Rwanda has five provinces (including Kigali city) and each province has a number of districts. In total, Rwanda has 499 health centers almost distributed equally in all districts of all provinces [21]. A health center in Rwanda is a primary health institution that offers preventive, curative, and rehabilitative health services and has between 10 and 20 nurses employed. In this study, the researcher intended to recruit 45 nurses into the study. However, due to possible attrition, the study intended to double the number of participants. 76 nurses participated in the pre-test and 56 finished the intervention and participated in the post-test. The study used simple random sampling to randomly choose two districts from each of the five provinces (including Kigali city) and randomly selected one health center from each chosen district, which makes up a total of 10 health centers. Moreover, based on inclusion criteria, researchers randomly intended to select 8 nurses at each health center to participate in this study. Thus, researchers requested the list of all nurses from the head of the health center and randomly selected potential participants. A nurse who was selected and did not fulfill the inclusion criteria or did not accept to participate in the study was randomly replaced by another nurse. Depending on inclusion criteria and willingness to participate, participants at each health center ranged from 4 to 8 nurses.

### Inclusion criteria

All nurses who worked at the health center, in consultation rooms, and reported proficiency in understanding the English language were potential participants in this study.

### Pre and post-data collection process

The potential participants who fulfilled the inclusion criteria and consented to participate in the study were requested to manage the cases of hypertension, depression, prostate cancer, and gastric cancer based on the local context of managing these disease conditions at the level of the health center (primary health care). Based on pilot study findings, the paper cases took approximately 20 min to complete on average. In the pre-test phase, a participant answered questions about non-communicable disease cases before using the VP case on a tablet. The questions of each disease case were on four separate pages, and a participant was given the next page after he/she had submitted the previous one and was not allowed to go back and change their previous answers. After completing the paper-based questionnaire, participants were provided with a tablet running Android and equipped with internet access for their CPD. The correct answers to the paper cases were not revealed to the participating nurses.

Additionally, each participant received a unique username and password to log in to the web-based VCS and access VP cases. The first author or a research assistant explained how to navigate the VP case on a tablet. After the short demonstration on navigating the VCS, participants were allowed to use the tablets whenever they wanted. The CPD was asynchronous, and participants worked on VP cases as their schedule permitted. After a participant completed the training, answered the questions of a VP case, and reviewed the feedback in the web-based VCS, they proceeded to take the post-test. During the work with the post-test paper cases, participants answered a similar questionnaire they answered in the pre-test, but this time, the identifiers of name, age, and residence of the paper case person were changed for distraction purposes. Participants used the same code on both the pre-test and post-test to help match the performance during analysis. All four VP cases followed a similar process: initially managing the case on paper (pre-test), then managing the VP case in the VCS, and finally, revisiting a new case on paper as a post-test.

**Fig. 3:**
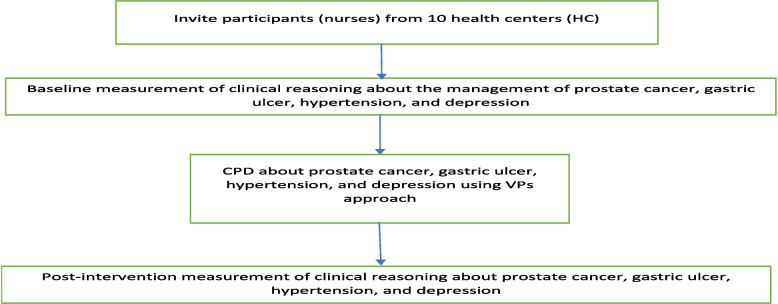
Illustration of pre-post data collection process

According to the pilot study's findings, the approximate time required for using a VP case was about 45 min per case. Because of other work responsibilities, we estimated that a participant could do one VP case per day, and therefore, data collection at each health center was performed over five to six days. The data collectors for the pre-post tests were stationed at the health center for five to six days while participants worked on the VP cases. Participants visited them for both pre-test and post-test sessions during this time. The demographic information of a participant was only filled in during the post-test test of the last case, and the analysis considered participants who finished all four VP cases. Data collection started on 13th March 2023 and ended on 8th June 2023. Figure 3, below illustrates pre-post test data collection.

### Data analysis plan

Data from the study were analyzed using the Statistical Software Package for Social Sciences (SPSS), version 26. Each questionnaire had a serial number, and during cleaning out of range and missing data, we double-checked the questionnaire. Participants' characteristics (demographic variables) were analyzed using frequencies and percentages. Participants’ scores on CPD courses using VP cases of hypertension, depression, prostate cancer, and gastric cancer in pre-test and post-test were dependent variables. Parametrical and non-parametrical assumptions relevant to tests were verified before using a relevant test. The data for scores of CPD using VP cases in both the pre-test and post-test were normally distributed, and the homogeneity of variances was not significant (*p*-value > 0.05). The study used a paired t-test to assess the effect of CPD through VP cases (explanatory variable) on the obtained score of managing a clinical case of hypertension, depression, prostate cancer, and gastric cancer. An alpha of 0.05 (2-tailed) was used to determine the significance of all statistical tests.

## Results

Initially, 76 participants were enrolled. Of these, 56 completed both the pre and post-tests as well as all four VP-cases and were considered for analysis. This number makes up a response rate of 76.7%. The participants who did not complete the study filled in the pre-tests and started the CPD through VP cases but reported insufficient time to finish all VP cases.

Please refer to Table 1 below for the demographic data of the included respondents.

**Table 1:** Demographic characteristics of participants (*n* = 56)

***Item***		***Frequency (%)***
Sex	Male	27 (48.2%)
	Female	29 (51.8%)
Age	Below 20	1 (1.8%)
	20—30	13 (23.2%)
	31—40	27 (48.2%)
	41 – 50	10 (17.9%)
	51 – 60	5 (8.9%)
Education level	Secondary (A2)	8 (14.3%)
	Diploma (A1)	34 (60.7%)
	Bachelor (A0)	13 (23.2%)
	Masters	1 (1.8%)
Place of work	Urban	14 (25%)
	Rural	42 (75%)
Professional experience	0 – 2 years	9 (16.1%)
	3 – 5 years	15 (26.8%)
	6 – 10 years	11 (19.6%)
	11 – 15 years	7 (12.5%)
	Above 15 years	14 (25%)

Further on, we could not detect any statistically significant differences in pre-test results resulting in the educational level of the nurses, see Table 2. However, there was a difference in post-test scores on gastric cancer based on the level of education. In the post-test of gastric cancer, participants with a diploma level scored significantly higher than participants with a secondary education level (0.039).

**Table 2:** Indicates the pre-test and post-test scores according to participants' education level

**Item**		**Freq(%)**	**Pre-test Mean(SD)**	***P*-value**	**Post-test Mean(SD)**	***P*-value**
Hypertension	Secondary (A2)	8	8.50 (1.3)	0.676	12.13 (1.2)	0.975
	Diploma (A1)	34	9.00 (1.9)		12.00 (1.8)	
	Bachelor (A0)	14	8.64 (1.5)		11.93 (2.6)	
Depression	Secondary (A2)	8	9.56 (2.7)	0.108	11.69 (2.3)	0.913
	Diploma (A1)	34	8.96 (2.6)		11.94 (1.5)	
	Bachelor (A0)	14	11.79 (1.9)		11.79 (1.9)	
Prostate cancer	Secondary (A2)	8	10.13 (1.9)	0.559	12.75 (1)	0.511
	Diploma (A1)	34	9.62 (2.1)		12.18 (1.8)	
	Bachelor (A0)	14	10.29 (2.3)		11.93 (1.3)	
Gastric cancer	Secondary (A2)	8	7.25 (1.3)	0.159	10.63 (1.7)	0.039
	Diploma (A1)	34	8.35 (2.1)		11.97 (1.7)	
	Bachelor (A0)	14	8.34 (2)		12.00 (1.6)	

 When it comes to participants' scores according to their area of work (urban or rural), the T-test analysis indicates no significant statistical difference based on participants’ working area, see Table 3.

**Table 3:** Survey score comparing participants' areas of working

**Item**		**Freq(%)**	**Pre-test Mean(SD)**	***P*-value**	**Post-test Mean(SD)**	***P*-value**
Hypertension	Urban	14	8.64 (2.1)	0.623	11.86 (2.1)	0.751
	Rural	42	8.90 (1.6)		12.05 (1.9)	
Depression	Urban	14	9.14 (2.1)	0.587	11.89 (1.6)	0.946
	Rural	42	9.57 (2.7)		11.86 (1.7)	
Prostate cancer	Urban	14	10.36 (1.5)	0.3	12.50 (1.4)	0.414
	Rural	42	9.69 (2.2)		12.10 (1.6)	
Gastric cancer	Urban	14	8.71 (2.2)	0.417	11.93 (1.4)	0.666
	Rural	42	8.21 (1.9)		11.74 (1.4)	

Regarding the outcomes from the VP-based training, a paired t-test demonstrates a significant increase in participants’ scores in managing clinical cases from the pre-test (before engaging in CPD using VP cases) to the post-test score (after engaging in CPD using VP cases). The *p*-value for all cases was < 0.001, indicating a significant improvement in clinical reasoning abilities after running the VP cases, see Table 4.

**Table 4:** T-test results comparing pre-test and post-test scores

Item	Min and Max score	Pre-test Mean(SD)	Post-test Mean(SD)	t(pdf)	*P*-value
Hypertension	0—15	8.84(1.7)	12(1.9)	-10.68(55)	< 0.001
Depression	0—15	9.46(2.5)	11.87(1.7)	-8.27(55)	< 0.001
Prostate cancer	0—15	9.86(2.1)	12.2(1.6)	-8.23(55)	< 0.001
Gastric cancer	0—15	8.34(1.9)	11.79(1.4)	-11.6(55)	< 0.001

## Discussion

This study aimed to determine the feasibility of using CPD through VP cases to improve nurses' clinical reasoning in primary health care. The study used pre-test and post-test paper cases to assess the clinical reasoning skills change before and after the intervention. At the study’s outset, 76 participants consented to participate, yet only 56 completed the study intervention, enabling their participation in the post-test assessment. While there is a possibility of attrition bias, as we lack information about whether the scores of those who dropped out of the study differed systematically from those who completed, we do not suspect that attrition was based on any systematic characteristic. This attrition issue may have been influenced by work responsibilities and other daily commitments, potentially hindering some nurses from completing the study.

The study results show that the mean score increased significantly from the pre-test to the post-test in all cases (*p* < 0.001). To the best of the authors’ knowledge, this study represents the initial endeavor to assess the feasibility of VP cases for CPD among nurses in primary health care. However, previous studies have utilized VP cases for training nursing students in their final years, and those who returned to University for further education, and these studies have consistently demonstrated the progression of clinical reasoning for the participants [11, 22–24].

The increase in scores from pre-test to post-test suggests a potentially positive contribution of VP cases for CPD of nurses at primary health centers, particularly in enhancing their clinical reasoning abilities. This claim is grounded in the observation that VP cases facilitate nurses in traversing through four stages of learning: concrete experience, reflective observation, abstract conceptualization, and active experimentation, aligning with Kolb's model of experiential learning [15]. The nurses who took part in this study all possessed experience in clinical reasoning and decision-making for patient management, but the features of the VP cases seem to stimulate their cognitive processes in terms of clinical reasoning abilities. For instance, in the history-taking section, there were multiple options available for a nurse to choose from; selecting the most appropriate questions to ask the virtual patient in this context reflects the process of applying clinical reasoning skills. Simmons defines clinical reasoning as "a complex process that uses cognition, metacognition, discipline-specific knowledge to gather and analyze patient information, evaluate its significance and weigh alternative action” ([16] p.1155), which seems to be improved in this study.

Using VP cases for practicing nurses is supported by Kolb’s assertion that learning involves the generation of knowledge through the transformation of experiences [15]. While traditional face-to-face and online CPD methods, which involve understanding principles and disease management protocols, remain valid, the VP cases approach seems to provide the additional benefit of exposing nurses to real-life-like cases they are likely to encounter in their daily practice. Using VP cases, they encounter these real-life-like cases in a learning environment where making an error cannot harm a patient, and they also have the opportunity to receive expert feedback from the VCS system.

The study findings did not show statistically significant differences based on participants’ demographic characteristics. For example, there was no difference in the performance scores between participants working in urban areas and those working in rural areas. While the difference in working areas could have been due to the availability and type of electronic devices or internet availability, we found that most often, the internet connectivity was equally available to all health centers involved, regardless of whether they were in a rural or urban area. In addition, we lent tablets and portable internet equipment to participants, and thus, they used similar devices with similar internet capabilities in both urban and rural areas.

The participants' education level was another variable that could have resulted in different scores. However, there were no statistically significant differences in most VP cases. This result may be based on the fact that secondary-level nurses had a long working experience compared to those with other education levels since the demand for nurses with diploma and bachelor degrees at district and referral health system levels is still high, which often makes them stay for a short time at primary health centers.

### Implication and recommendations from the study

The literature indicates that VP cases are predominantly used in pre-service training of health care providers and seldom for those who have started practicing. The reluctance to utilize VP cases for CPD may stem from the assumption that practicing nurses primarily rely on on-site learning to engage with patients and learn from their more experienced colleagues. While on-site learning can be effective and adequate for developing the clinical reasoning skills of practicing nurses, it may have limitations in certain contexts, such as health centers in Rwanda. A newly graduated nurse working at health centers in Rwanda is typically assigned to the consultation room. In this setting, the nurse operates independently and is responsible for triaging patients, managing various medical conditions at health centers, or referring patients to higher-level healthcare facilities, such as district hospitals, where they can consult physicians or specialized healthcare providers. This working environment restricts the opportunities for nurses at health centers to interact with colleagues, making VP cases a valuable tool to help them stay up-to-date with managing diverse medical cases.

Another rationale for using online training through VP cases for CPD at health centers is the transition from communicable to non-communicable diseases as the predominant health concern [2]. Infectious diseases, such as malaria in endemic regions, often have well-defined protocols for their screening and management. However, establishing clear screening protocols for every non-communicable disease at the health center level can be challenging. In such a scenario, VP cases can facilitate nurses at this level of care to engage with a variety of non-communicable disease cases, thereby enhancing their clinical reasoning skills in terms of timely diagnosis, management, or appropriate referrals. Furthermore, VP cases are provided in the form of online CPD, which offers a number of advantages over physical CPD. For example, online VP cases enable nurses to engage in CPD without leaving their workplace, which necessitates transportation and more time to attend in-person meetings. Online solutions also offer the possibility to participate in training anytime, facilitating clinical nurses to participate in CPD.

### Limitations of the study

While the study hypothesized that CPD using VP cases would improve nurses’ ability to reason about the management of clinical cases related to hypertension, depression, prostate cancer, and gastric cancer, it is important to note that the study did not account for other potential confounding variables: External factors, such as seeking assistance from colleagues and consulting multiple information sources, which might have enhanced comprehension of case management, were not accounted for or controlled for in the study. However, all participants were instructed not to consult colleagues or other educational material during the study. Sampling bias could also affect this study, as it excluded participants who believed they could not read and comprehend English. Even though English is the official language in Rwanda, some nurses have been trained in French and thus might use French as their main language, not English. Furthermore, despite differences in identifying information among the VP cases in the pre-test, VP cases on the tablet, and post-test, we acknowledge that the nature of the study, which involves running cases within the same illness domain during pre-CPD case management (answering pre-test questions) and managing the VP case on a tablet, could have contributed to improved case management in the post-assessment.

### Conclusion and recommendation

This study shows that VP cases enhance a deliberate learning process for CPD and enable the nurses to reflect on how they managed the cases, thus improving their clinical reasoning skills. This study strongly recommends the incorporation of VP cases in the continuous professional development of nurses at the primary health level (health centers). This is especially pertinent in a context where nurses are required to perform diagnostic processes similar to those employed by physicians, like in Rwanda.

## Data Availability

The datasets used during the current study are not available due to a lack of ethical authorization for sharing raw data. However, they can be obtained from the corresponding author on reasonable request.

## Abbreviations

CPD: Continuous Professional Development
VP cases: Virtual Patient Cases
